# Space Competition and Time Delays in Human Range Expansions. Application to the Neolithic Transition

**DOI:** 10.1371/journal.pone.0051106

**Published:** 2012-12-10

**Authors:** Neus Isern, Joaquim Fort, Marc Vander Linden

**Affiliations:** 1 Departament de Prehistòria, Universitat Autònoma de Barcelona, Cerdanyola del Vallès, Spain; 2 Complex Systems Lab, Departament de Física, Universitat de Girona, Girona, Catalonia, Spain; 3 Institute of Archaeology, University College London, London, United Kingdom; University of Florence, Italy

## Abstract

Space competition effects are well-known in many microbiological and ecological systems. Here we analyze such an effect in human populations. The Neolithic transition (change from foraging to farming) was mainly the outcome of a demographic process that spread gradually throughout Europe from the Near East. In Northern Europe, archaeological data show a slowdown on the Neolithic rate of spread that can be related to a high indigenous (Mesolithic) population density hindering the advance as a result of the space competition between the two populations. We measure this slowdown from a database of 902 Early Neolithic sites and develop a time-delayed reaction-diffusion model with space competition between Neolithic and Mesolithic populations, to predict the observed speeds. The comparison of the predicted speed with the observations and with a previous non-delayed model show that both effects, the time delay effect due to the generation lag and the space competition between populations, are crucial in order to understand the observations.

## Introduction

Reaction-diffusion equations have been widely applied to model biological and cross-disciplinary systems where there is an expanding front, such as in population dispersals, tumor growth or virus infections [1]. In this paper we develop a reaction-diffusion model with time delay and space competition that we apply to predict the spread of the Neolithic transition in Europe.

The Neolithic transition is the change from a foraging way of subsistence (Mesolithic) to a farming lifestyle (Neolithic). Neolithization is considered a crucial process in human history as it involved a global socioeconomic change, and thus has been subject of numerous studies from different disciplines, such as archaeology, anthropology, genetics or mathematical modelling [2]. In Europe, the analysis of datings for early Neolithic sites shows that farming arrived from the Near East, the Fertile Crescent, and spread throughout the continent between 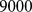 and 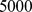 years ago [3], [4].

The mechanisms for this spread have been traditionally regarded from two contrasting points of view. The demic diffusion model considers that the Neolithic expansion was mainly a demographic process, i.e. farming was spread by the dispersion of farmers [5]. On the other hand, the cultural diffusion model assumes that the adoption of the new culture and techniques by the indigenous populations is the main mechanism for the Neolithic expansion, entailing the spread of ideas rather than individuals [6].

There are archaeological analyses of Neolithic datings that support the idea of a mostly demic diffusion [3], [7]–[9], but it is specially relevant that several recent genetic studies also agree with the demic diffusion scenario, both from the analysis of modern European populations [10], [11], as well as from the DNA of ancient remains [12]–[14].

Thus, assuming that Neolithization was basically a demic process, one can apply reaction-diffusion equations to model its expansion in Europe. The first attempt to mathematically model the Neolithic transition in Europe was led by Ammerman and Cavalli-Sforza in 1973 [15] applying the wave of advance model initially proposed by Fisher to predict the spread of advantageous genes [16]. Since then, other authors have developed more complex reaction-diffusion models to try to better explain the Neolithic expansion, for example, by including the effect of the time delay between successive migrations [3], [17], [18]. Fisher's model considers that there is no time delay between two successive migrations, however, when modelling human beings it is reasonable to assume that children will stay with their parents until they reach adulthood and migrate to form their own families. Thus there is a time span between the migration of the parents and that of their children that, when taken into account, yields slower speeds than Fisher's model, and which are consistent with the observations for the Neolithic transition [3].

Besides human population dispersals, there exist other biological systems in which the inclusion of a time delay between migrations has also proved to be of great importance to predict rates of expansion, such as the range expansion of some avian species [19] (where the time delay is due to the reproduction time) or viral infections of bacteria [20] or mammalian cells [21] (where the time delay there would correspond to the elapsed time between the virus adsorption and the release of its progeny).

Another important effect that neither Fisher's model nor the time-delayed models take into account is the space competition between the colonizing farmers (Neolithic populations) and the indigenous hunter-gatherers (Mesolithic populations). Space competition between populations has been widely studied in ecological [22], [23] and microbiological systems [24]. On the spread of human populations, the interaction between different cultural groups can also have an important effect. In recent years, several authors have developed reaction-diffusion models that tackle the interaction between farmers and hunter-gatherers when studying the Neolithic transition [25]–[28]. Here we will focus on the models developed in references [27], [28] that were applied to predict the observed slowdown of the Neolithic transition in Northern Europe in terms of the space competition between Neolithic and Mesolithic populations. Archaeological literature points out that this slowdown may be caused by an increase on the Mesolithic population density near the North Sea [29]–[31]. Other theoretically possible causes for the slowdown, such as the genetic adaptation of crops to the different climatic conditions, have been considered negligible as, according to Coward et al. [32], apparently the crops that were not productive enough were dropped for the other more productive ones. Then, we have a system in which one population (Neolithic) colonizes an already populated region where the indigenous population density (Mesolithic) is not homogeneously distributed. The interaction between the two populations modifies the rate of spread of the invading population. It has been shown that the space competition between the two populations can be mathematically modelled by considering that the interaction has two effects. First, a limiting effect on the Neolithic population (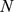) growth dynamics, due to the competition for space and resources. So, besides the self-limiting term usually introduced in the logistic growth equation (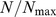) [22], one must also add a limitation term due to the fraction of space already occupied by the Mesolithic populations (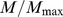). As shown in Ref. [27], then the usual logistic growth function 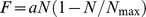 is replaced by 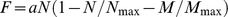 (see Materials and Methods). On the other hand, the dispersion is also affected by the presence of Mesolithic populations and can be described through a non-homogeneous dispersion probability depending on the Mesolithic population density 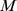 at each direction. In this way, the probability to move towards a certain direction depends on the space available (or free space 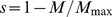) in that direction [27]. Using these ansätze one can predict modifications on the front speed dependent on the distribution of the Mesolithic populations [27], [28].

However, these previous models do not take into account the effect of the time delay between successive migrations (and neither do the other models with interaction mentioned above [25], [26]). In this paper we want to develop a more general model including both effects, namely, the interaction between populations and the time delay between successive migrations. We will apply this model to predict the slowdown of the Neolithic transition in Europe and compare the results with the observations from a comprehensive Early Neolithic database for Europe containing 902 Early Neolithic datings (included as Table S1). In the next sections we will introduce a model where both effects (the interaction between populations and the time delay) are considered, and see that it can give a better account of the observed front speeds.

## Results

### Observed front speeds

**Figure 1:**
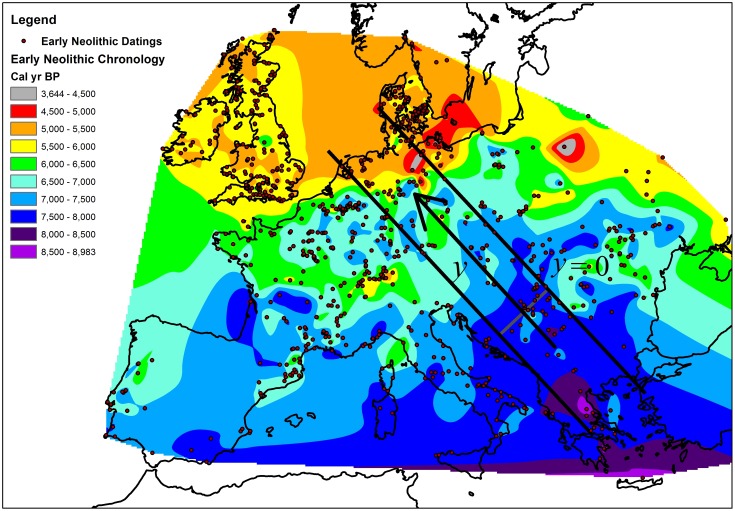
Chronology of arrival times of the Neolithic transition in Europe. The circles correspond to the 902 datings (included as Table S1) used for this natural neighbor interpolation. The delimited corridor defines the region studied here, where the Neolithic expansion took place mainly in the 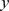 direction. The origin of the 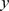 coordinate is also defined on the map.

Figure 1 shows an interpolation of 902 Early Neolithic datings (included as Table S1), obtained using a natural neighbor interpolation method [33], and the location of the archaeological sites. This map gives an idea of how the Neolithic spread throughout the continent. From the interpolation map one can already qualitatively perceive a slower rate of expansion at the Atlantic coast, obvious by the fact that the distance advanced in 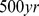 (distance between isochrones) is smaller there than at lower latitudes.

**Figure 2:**
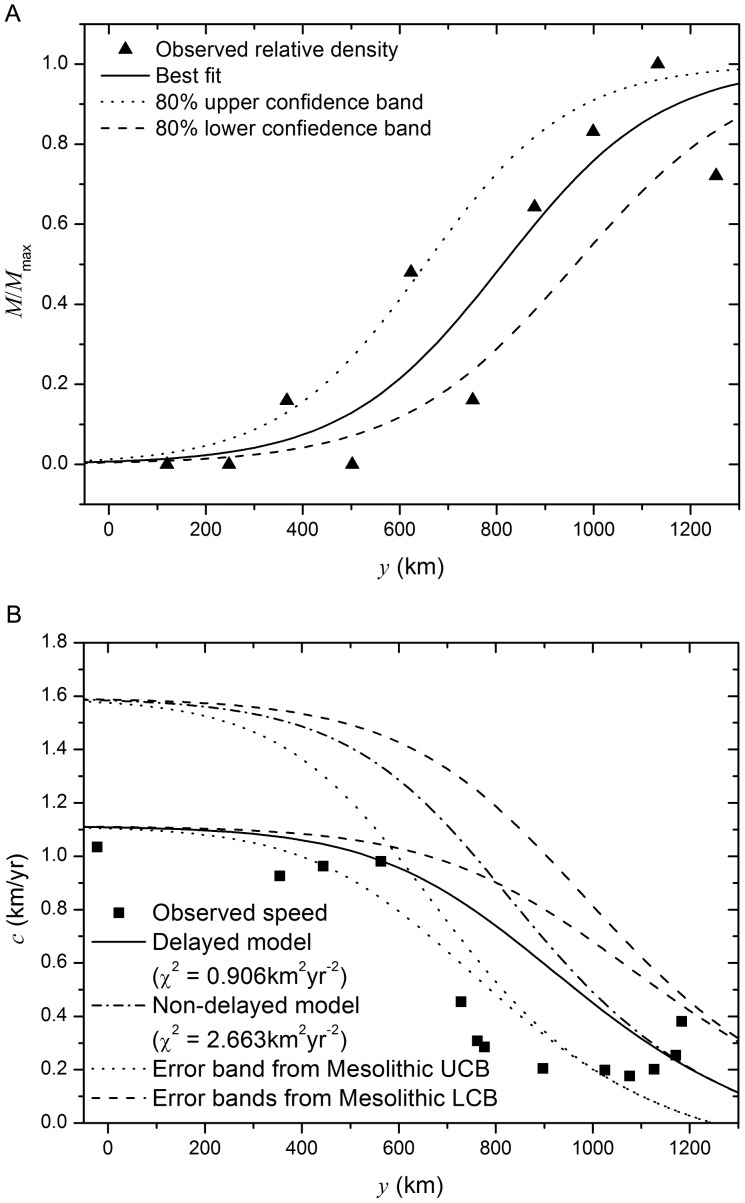
a) Variation of the Mesolithic population density in the region of study. The triangles correspond to the archaeological data and the lines to the best fit (solid line) [28], and the upper and lower 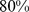 confidence bands (dotted and dashed lines respectively). (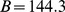 and 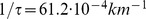 for the best fit, solid line, 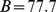 and 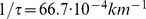 for the upper band, dotted line, and 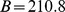 and 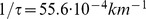 for the lower band, dashed line.) b) Estimated and predicted speeds for the slowdown of the Neolithic transition. Symbols (squares): measured front speed from archaeological data for the region delimited in Fig. 1 (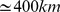 wide) and using 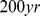 intervals. Lines: Prediction from a time-delayed reaction diffusion-model with space competition between populations (solid line) and from a non-delayed model with space competition (dash-dotted line) when considering the best fit for the Mesolithic data, and when using the upper and lower bands for the Mesolithic population density (dotted and dashed lines respectively) from (a). (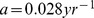, 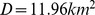 and 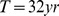.)

**Figure 3:**
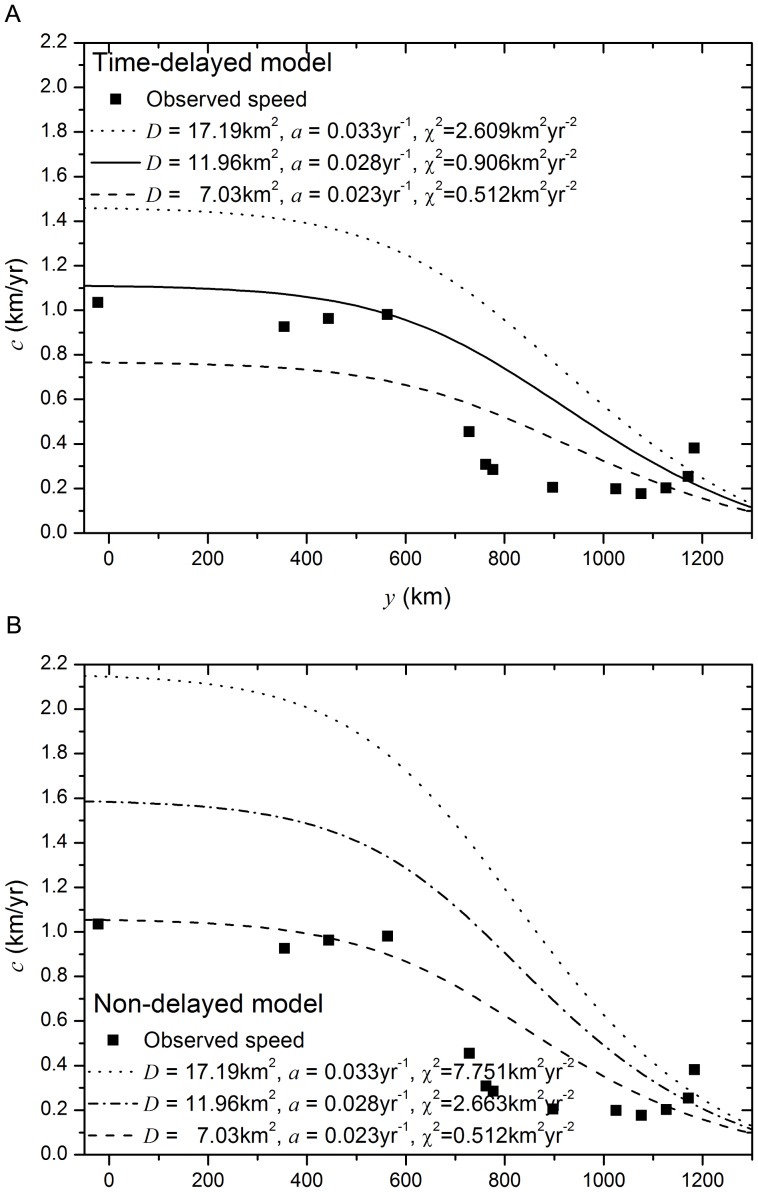
Parameter sensitivity of the models. Symbols (squares): Measured front speed from archaeological data. Lines: Upper and lower error bands (dotted and dashed lines respectively) predicted when considering the 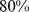 C.L. intervals for 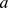 and 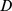 for (a) the time-delayed model and (b) the non-delayed model. The solid line in (a) and the dash-dotted line in (b) correspond to the mean prediction, i.e., they are the same as in Fig. 2b. (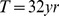, 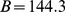 and 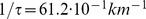).

In Fig. 1 we use 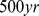 intervals for clarity, but we calculate the front speed from the interpolated results every 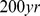. The front speed is calculated using a geometrical method (see Materials and Methods) for a corridor that goes from the Balkans to the North Sea, as indicated by the straight lines in Fig. 1. The axis of this corridor roughly corresponds to one of the main known axis of diffusion of farming across Europe and encompasses, in the South-East, some of the earliest occurrences of farming in Europe, and in the North-West some of the latest (the latest are located in Northern Scandinavia, which is actually excluded here). The calculated front speeds are represented in Figs. 2b, 3a and 3b (squares), with the first value corresponding to the period between 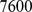 and 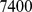 Cal yr BP; earlier periods have been left out in order to avoid the sea travel effect near the Mediterranean. (Note that this first value is located at 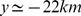, rather than 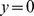. The origin of the 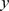 coordinate, 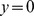, was defined as the position of the first archaeologically-measured value in references [27], [28], but as we are using a newer database here, the position of the first value does not lie in the same position as before. However, we still conserve the same origin as with the older database, for the sake of comparability.)

The measured front speeds show that the expansion was, at first, approximately constant at a rate of about 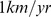 (at central Europe), which agrees with the mean front speed values obtained by Gkiasta et al. [8] and Pinhasi et al. [3], but later there is a clear slowdown on the expanding speed as Northern regions are reached, mostly between 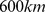 and 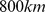 (see, e.g., Fig. 2b).

The results shown in Figs. 2b, 3a and 3b have been calculated using 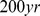 intervals, but changing the interval duration does not change the observed results significantly, and neither does changing the width of the studied regions to a wider corridor (see Fig. S1). Changing the interpolation method (Fig. 1 corresponds to a Natural Neighbor interpolation) modifies slightly the values of the front speed, but not significantly enough as to change the conclusions in this paper (see Figs. S2 and S3).

### Mathematical model

When considering both effects (namely, the interaction with the Mesolithic populations and the time delay between migrations) the reaction-diffusion equation that describes the evolution at the leading edge of the Neolithic front (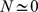) can be expressed as follows (see Materials and Methods),
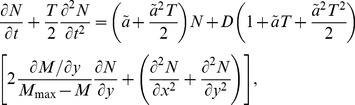
(1)where 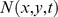 is the Neolithic population density at position 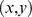 and time 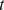, 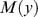 is the Mesolithic population density upon the arrival of the Neolithic transition (for simplicity 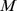 is considered to change only along the 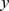 direction, as indicated in Fig. 1), 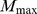 is the maximum Mesolithic population density in the region, 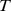 is the Neolithic generation time, 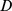 the Neolithic diffusion coefficient and 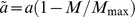 is the modified growth rate for the Neolithic populations due to the presence of Mesolithic populations (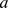 being the Neolithic intrinsic growth rate).

The interaction with Mesolithic populations is clearly taken into account in Eq. (1) by the use of 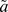 instead of 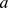 (limiting the population growth due to the competition for space and resources) and the term proportional to 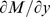 where the Mesolithic population density 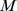 appears explicitly (backwards advection term that hinders the expansion rate due to encountering Mesolithic populations). Taking into account the time delay entails the appearance of the second-order derivatives in time [17], which in the reaction-diffusion equation (1) correspond to the second term on the left-hand side and the terms proportional to 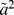 on the right-hand side (see Materials and Methods for the detailed derivation). Conversely, when only taking into account the space competition, but not the time delay (such as in Ref. [28]) the reaction-diffusion equation would be
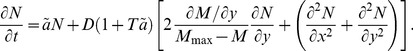
(2)


The predictions for the Neolithic expansion obtained from the new reaction-diffusion equation (1) will be calculated below with the following equation for the front speed along the direction 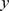 (see Materials and Methods for details)
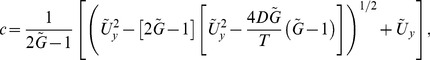
(3)where we have defined
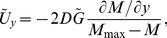
(4)

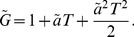
(5)


### Predicted Neolithic front speeds and parameter sensitivity

In order to apply Eq. (3) to the Neolithic transition we need to assign realistic values the Neolithic parameters and to define the equation 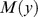 for the Mesolithic population density. For the Neolithic populations we use the following parameter values obtained from preindustrial farming populations (see Materials and Methods) 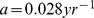 (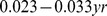 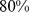 C.L. interval) [34], 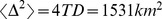 [35] (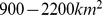 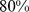 C.L. interval [3]) and 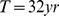 [36] (the value of 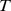 has little effect on the front speeds [17]).

The distribution of the Mesolithic population density upon the arrival of the Neolithic populations is unknown, but it is reasonable to assume that this will be proportional to the density of archaeological sites [37]. In Ref. [28] an estimation of the density of Mesolithic sites was performed from archaeological data [38], obtaining an increase of the population density at Northern latitudes (triangles in Fig. 2a). The best fit to the Mesolithic data along the direction 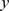 is given by the 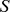-shaped function
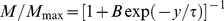
(6)with 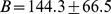 and 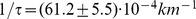 (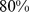 C.L. intervals). Fig. 2a shows the best fit (solid line) and the 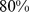 confidence bands (dashed and dotted lines). (Note that the lower band in Fig. 2a corresponds to using 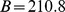 and 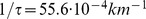 and the upper band to 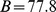 and 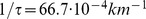.)

In Fig. 2b we show the front speeds predicted when applying the time-delayed model developed here, Eq. (3), as well as the previous non-delayed model given by Eq. (2), namely [28]

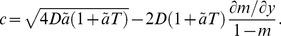
(7)Comparing first the results when applying mean parameter values (solid and dash-dotted lines in Fig. 2b) we see that, for both models, the predicted font speed at central Europe (i.e. the first 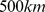) is mostly constant, but while the time-delayed model (solid line) predicts a front speed similar to the observations (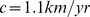 at 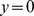) for this region, the non-delayed model (dash-dotted line) predicts a significantly faster speed (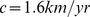 at 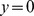). At Northern regions, both models predict a significant slowdown on the front speed similar to the observations, though somewhat less abrupt, with the non-delayed speeds always faster than those from the archaeological data; so the time-delayed model does also provide a better approximation for this region. In fact, if considering the whole range of distances, one obtains that 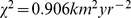 for the time-delayed model, Eq. (3), while for the previous non-delayed model, Eq. (7), the fit is poorer, with 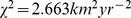.

Besides the prediction when considering the best fit to the Mesolithic population density, Fig. 2b also shows the predicted front speeds when considering 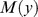 defined by the upper and lower confidence bands in Fig. 2a. We can see that changing the values of the parameters 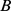 and 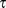 in Eq. (6) basically affects the abruptness of the slowdown, while the predicted front speeds near the origin are mostly unchanged. Then, for a more steep increase in the Mesolithic population density taking place at a southern region (dotted line in Fig. 2a, corresponding to 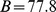 and 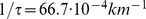), the predicted front speed also shows a more abrupt change (dotted lines in Fig. 2b). In fact, we see from Fig. 2b that this case would offer a better fit to the archaeological data, with 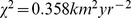 for the time delayed-model, Eq. (3), and 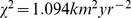 for the previous non-delayed model, Eq. (7). (All results in Fig. 2b have been calculated using the mean values of the Neolithic parameters 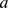, 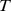 and 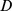.)

In Figs. 3a and 3b we analyze the errors introduced in the predicted speeds due to the uncertainties on the Neolithic parameters. The upper and lower bounds in Fig. 3 have been calculated using the extreme values of the 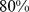 C.L. intervals for the initial growth rate 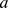 and the diffusion coefficient 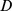. We see that, in this case, varying the Neolithic parameters modifies the predicted front speeds specially for the Southern regions (low values of 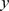). Also, it is in this Southern region where there is a more significant difference between the predictions from the time-delayed model (Fig. 3a) and the non-delayed one (Fig. 3b). We see in Fig. 3a that the time delayed-model developed here, Eq. (3), predicts a narrower window of front speeds as compared to the non-delayed model (Fig. 3b). At Southern latitudes (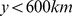), the archaeologically-measured speeds (squares) lie in the middle of the range predicted by the new delayed model (Fig. 3a) but only at the lower end of the range predicted by the previous non-delayed model (Fig. 3b). But even in the most favorable case for the non-delayed model (minimum values of 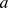 and 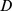), the prediction is not better that the one from the delayed model for the same set of parameters (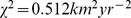 for both models in these conditions). Thus, even when considering the uncertainties on the Neolithic demographic parameters, the time-delayed model, Eq. (3), provides a better prediction of the observed data.

## Discussion

Previously, the Neolithic transition in Europe was modelled by taking into account the role of a delay time due to the generation lag time [3], [17]. Here we have also included the effect of the space competition between Neolithic and Mesolithic populations. We see from the results (Figs. 2b and 3) that the new model combining both effects, when applied to predict the slowdown of the Neolithic transition, provides a good approximation to the observed slowdown on the front speed.

In this paper we have compared the results from our models to a newer archaeological database (included as Table S1) than the one used in previous studies dealing with the slowdown of the Neolithic at Northern Europe [27], [28] (the older database can be found in Ref. [3]). This new database is more complete (902 European Early Neolithic datings, versus 765 Early Neolithic datings for Europe and the Near East) and has been carefully prepared and audited as to provide a reliable vision of Early Neolithic in Europe. Details on the data selection can be found in Ref. [39] and the entire database will be published as Ref. [40]. Consistent with the older database, a slowdown is observed (Fig. 1). The analysis of this newer and more detailed database for the corridor in Fig. 1 provides front speed values (squares in Figs. 2–3) that differ significantly from the predictions made by the previous model (see, e.g., the three upper lines in Fig. 2b). This problem is solved here by including the effect of the time delay between successive migrations.

The need of space-competition models when modelling the Neolithic transition has been noted only recently. It is true that the Mesolithic population densities at the time were rather low at most of the continent. In fact, according to Shennan and Edinborough [41], the Mesolithic population density just before the Neolithization process was lower than in previous periods (and much lower than the Neolithic population densities reached upon the consolidation of the Neolithic culture) (see also [42]). The estimation of the Mesolithic population density for our region of interest performed in Ref. [28] (triangles in Fig. 2a) also yields very low values of Mesolithic population density at central Europe (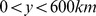). Certainly, low Mesolithic population densities would have had little effect on the front speed, which is consistent with the mostly constant front speed both measured from he Neolithic database (squares in Fig. 2b) and predicted by the models at the first 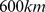 of the studied region. Thus, except in Northern Europe, it seems reasonable to neglect the interaction between Mesolithic and Neolithic populations when modelling at large scales. Conversely, in the particular case studied here (Northern Europe), the space competition between populations seems to be of utmost importance. Indeed, taking into account the presence of non-homogeneously distributed Mesolithic populations, and the interaction of these indigenous populations with the Neolithic individuals, yields in our model a slowdown on the expanding front, similar to the observations. In addition, we have seen (Fig. 2b) that the way in which the slowdown predicted by the models takes place depends highly on the shape of the function for the Mesolithic density 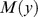. The results have been calculated taking into account the uncertainties in the parameters 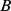 and 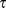 in Eq.(6), and we have seen that while the best fit to the Mesolithic data obtained in Ref. [28] provides a reasonable account for the measured variation in the front speeds, an earlier and more abrupt increase in the Mesolithic population density (such as when considering the upper 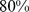 confidence band from Fig. 2a) would be able to provide a better fit to the observed front speeds.

On the other hand, when modelling dispersions of human populations there is a time delay between the migration of the parents and the time in which their children can migrate away and create their families. Fisher-type models do not take into account such a delay. When included in the mathematical models (by including second-order time derivatives at the reaction-diffusion equation) this delay yields significantly slower front speeds, as can be easily seen by comparing, e.g., the results from the delayed and non-delayed models in Fig. 2b. When comparing the mean predictions from the models (solid and dash-dotted lines in Figs. 2b and 3) with the archaeological data (squares) we see that, in the particular case studied here, taking into account the time delay can provided a significantly better prediction of the measured front speed. This is specially obvious for the first 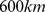 of the studied region where the mean error of the prediction is about 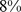 for the delayed model while for the non-delayed model the mean error is 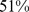. But also when considering the whole region, the time-delayed model provides a better prediction for the front speed, as shown by the lower value of 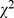 obtained when comparing the archaeological data with the models.

When working with archaeological and anthropological data, the uncertainties in the parameters and measurements can have an important effect on the predicted results. We have discussed above how taking into account the uncertainties of the parameters defining the function for the Mesolithic affects the predicted results, but in this paper we have also studied the importance of the uncertainties in the demographic parameters 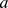 and 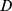. Taking into account the extreme values of the 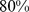 C.L. interval for 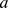 and 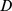 provides a fairly wide range of possible predicted speeds, specially at the first 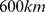. In the case of the time-delayed model developed here, for this initial region, the range of predictions is mostly centered around the archaeological estimates. The non-delayed model, on the other hand, provides a wider window of possible results, from speeds marginally consistent with the observations in the slower case, to speeds up to a 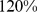 faster that the observations for the first 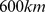. However, even though the previous non-delayed model could be considered marginally consistent with the measured data given certain conditions of low diffusivity and population growth (i.e., due to the parameter uncertainties), this prediction is not better that the one from the delayed models in the same conditions. Thus, even taking into account the effect of the uncertainties, the time-delayed model can still provide a better prediction of the measured front speeds for the slowdown of the Neolithic transition.

Therefore, the new model developed here with space competition and time delay effects provides a better prediction of the measured slowdown of the Neolithic transition than previous approximations taking into account only one of these effects [3], [27], [28]. So, in this paper we see that (i) reaction-diffusion models taking into account the space competition with indigenous groups (or species) can predict variations in the front speed (a deceleration in this case) related with inhomogeneities on the indigenous population distribution and (ii) that including the time delay effect due to a generation lag can provide much better descriptions of observations.

In future work, it would be of interest to perform numerical simulations to check that the theoretical front speed predicted here is actually obtained within the domain, given the finite time and length scales imposed by the archaeological dates and geographical region analyzed.

## Materials and Methods

### Speed from archaeological data

In this paper we have compared the results from our models with archaeological speeds estimated from an Early Neolithic database with 902 sites, which is included as Table S1 (details on the data selection can be found in Ref. [39]). The database includes information on the location, dating, dating method and dated material. To calculate the front speed in Fig. 2 we have calibrated the 902 dates from the database using CALIB Radiocarbon Calibration 5.0.1 software [43], and interpolated the calibrated dates using a natural neighbor interpolation system [33] with ArcGIS 10. Fig. 1 shows the chronology of arrival times for the Neolithic transition thus obtained. Setting the isochrones at 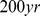 intervals, for each 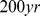 interval we have defined a polygon delimited by two isochrones and the limits of the studied region. Computing the area of each of these polygons we know the region covered by the Neolithic population expansion during each 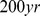 interval within the studied region. Thus, assuming that the expansion took place mainly in the direction 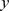 indicated in Fig. 1 we can estimate the mean front speed from the area, the width of the considered region (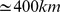) and the time interval (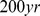) with the following expression
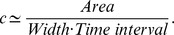
In order to plot these results, for each area we have also calculated the 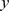 coordinate of its centroid (by using the corresponding built-in function from ArcGIS). In this way, for each 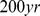 interval we have a value of 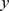 and a mean front speed.

We have also applied the same method to estimate front speeds when considering 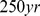 intervals, as well as for a wider region (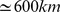). They all provide approximately the same results as can be observed in Fig. S1. We have also preformed similar calculations using a kringing interpolation method [44] with a spherical semivariogram (see map and estimated front speeds in Figs. S2 and S3). In this case the front speeds are slightly different than with the natural neighbor interpolation method, but the conclusions for this paper remain unchanged.

### Mathematical model: Analytic front speed

The reaction-diffusion model developed here, Eq. (1), can be obtained from the following evolution equation that gives the Neolithic population density after a time interval 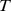 (a generation) due to the reaction (population growth) and dispersal processes [28]

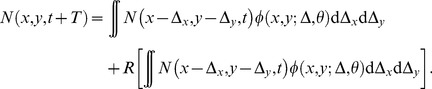
(8)The function 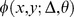 is the dispersion probability distribution that provides the probability for the Neolithic individuals to move from the position 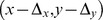 to 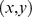 during one generation. We have defined this probability distribution as dependent on the density of Mesolithic populations 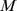 present at each position. Assuming that 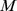 varies only on the direction 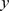 we have [27]

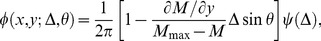
(9)where 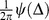 is the probability distribution corresponding to the dispersion in an unpopulated space, with all directions equally probably, 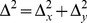 and 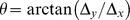. The function 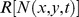 corresponds to the reproduction process and gives the increase in population density due to the birth-death balance during one generation. 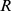 can be expressed as a Taylor series of the population growth function 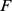,
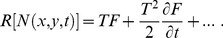
(10)We have defined the population growth function 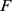 similarly to the logistic growth equation, but taking into account the additional limiting effect to the Neolithic population growth due to competition for space and resources with the Mesolithic populations [27]

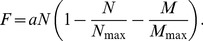
(11)


Linearizing and Taylor-expanding Eq. (8) up to second order in time and space, we obtain the reaction-diffusion equation that describes this system including the interaction between populations and the time delay effect, i.e., Eq. (1). Due to the linearization, Eq. (1) is valid only at the leading edge of the front (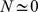), which is where we want to compute the expansion speed. We calculate the front speed by assuming that for 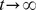 the front is locally planar, with the local speed 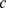 parallel to the 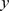 direction when 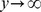. Then, we look for constant shaped solutions with the form 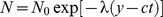 when 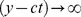. As 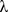 has to be real, this yields the following constraint for the front speed
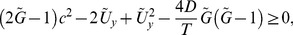
(12)where 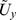 and 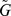 are defined in Eqs. (4) and (5). As the speed is a positive value, from Eq. (12) one obtains that the real speed is 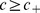,with 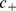 the positive root to the quadratic equation obtained when the equality in Eq. (12) holds. From variational analysis [45] one can also obtain an upper bound that happens to be the same value, thus 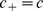 is the exact front speed for a system described by the reaction diffusion equation (1), and this front speed can be expressed mathematically by Eq. (3) in the Results section.

### Parameter values

To predict the Neolithic front speed we have used the following Neolithic parameter values: 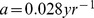 (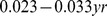 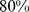 C.L. interval) [34], 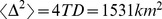 [35] (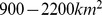 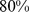 C.L. interval [3]) and 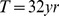 [36] (the value of 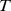 has little effect on the front speeds [17]). All these ranges have been estimated from modern ethnographical data of preindustrial populations as we summarize below.

The range above for the intrinsic growth rate 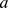 was estimated in Ref. [34] from data on the evolution of the population number for human populations established in previously unpopulated space (Pitcairn [46], Furneaux [46] and Tristan da Cunha [47] islands, and the fist colonization of the United States). The intrinsic growth rate has also been estimated by Guerrero et al.[48] directly from archaeological remains based on the rise in fertility (which was detected due to the rise in the proportions of immature skeletons in early Neolithic cemeteries), obtaining 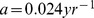, which lies within the range above (that was estimated from modern pre-industrial farming populations).

The other two parameters used here, 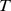 and 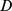, were estimated from ethnographical data from the agriculturalist Majangir people in Ethiopia. The generation time 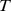, defined as the mean age difference between parents and one of their children (not necessarily the eldest), was estimated previously (see note [24] in reference [36]). The diffusion coefficient has been calculated from the expression 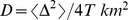 [17], and the mobility range was estimated in Ref. [3] from the distance moved by individuals during one generation for three Majangir groups [35].

To include the space competition between populations, we need the Mesolithic population density upon the arrival of the Mesolithic front. The actual Mesolithic population density is unknown, but as only the relative density is necessary, it is reasonable to assume that it will be proportional to the density of archaeological sites [37]. The relative density of Mesolithic sites was estimated in Ref. [28] using data from the INQUA database [38], for a region 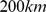 wider than the one delimited in Fig. 1. The best fit to these data yield the following expression 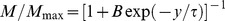, with 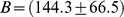 and 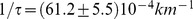. The behavior of this function corresponds to a relative Mesolithic populations density 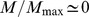 at 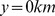, which increases following a 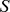-shaped curve with 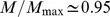 at 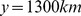, as shown in Fig. 2a.

## Supporting Information

Table S1 Information about 902 Early Neolithic sites: latitude/longitude, radiocarbon date, calibrated date, and additional archaeological information [39].
(XLS)

Figure S1Front speeds estimated from the archaeological data interpolated with a natural neighbor method and using 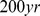 (squares, as in Figs. 2b, 3a–b) and 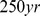 (triangles) intervals for a corridor 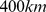 (see Fig. 1) wide, and 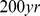 (stars) and 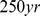 (crosses) intervals for a corridor 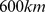 wide. The solid line corresponds to the delayed model with space competition (Eq. (1)), i.e., it is the same as the solid line in Figs. 2b and 3a.(TIF)

Figure S2Chronology of the Neolithic expansion calculated with a kringing interpolation method with a spheric semivariogram. The circles correspond to the 902 datings used for this interpolation. The delimited corridor defines the region studied here, where the Neolithic expansion took place mainly in the direction 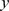. The origin of the 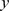 coordinate is also defined on the map.(TIF)

Figure S3Front speeds estimated from the archaeological data interpolated with a kringing method and using 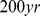 (squares) and 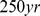 (triangles) intervals for a corridor 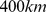 wide (see Fig. 1). The solid line corresponds to the delayed model with space competition (Eq. (1)), i.e., it is the same as the solid line in Figs. 2b and 3a.(TIF)
