# Supplementary figures and images for: Space Competition and Time Delays in Human Range Expansions. Application to the Neolithic Transition
Source: PLoS One. 2012 Dec 10;7(12):e51106. doi: 10.1371/journal.pone.0051106 (PMC3519538; doi:10.1371/journal.pone.0051106)

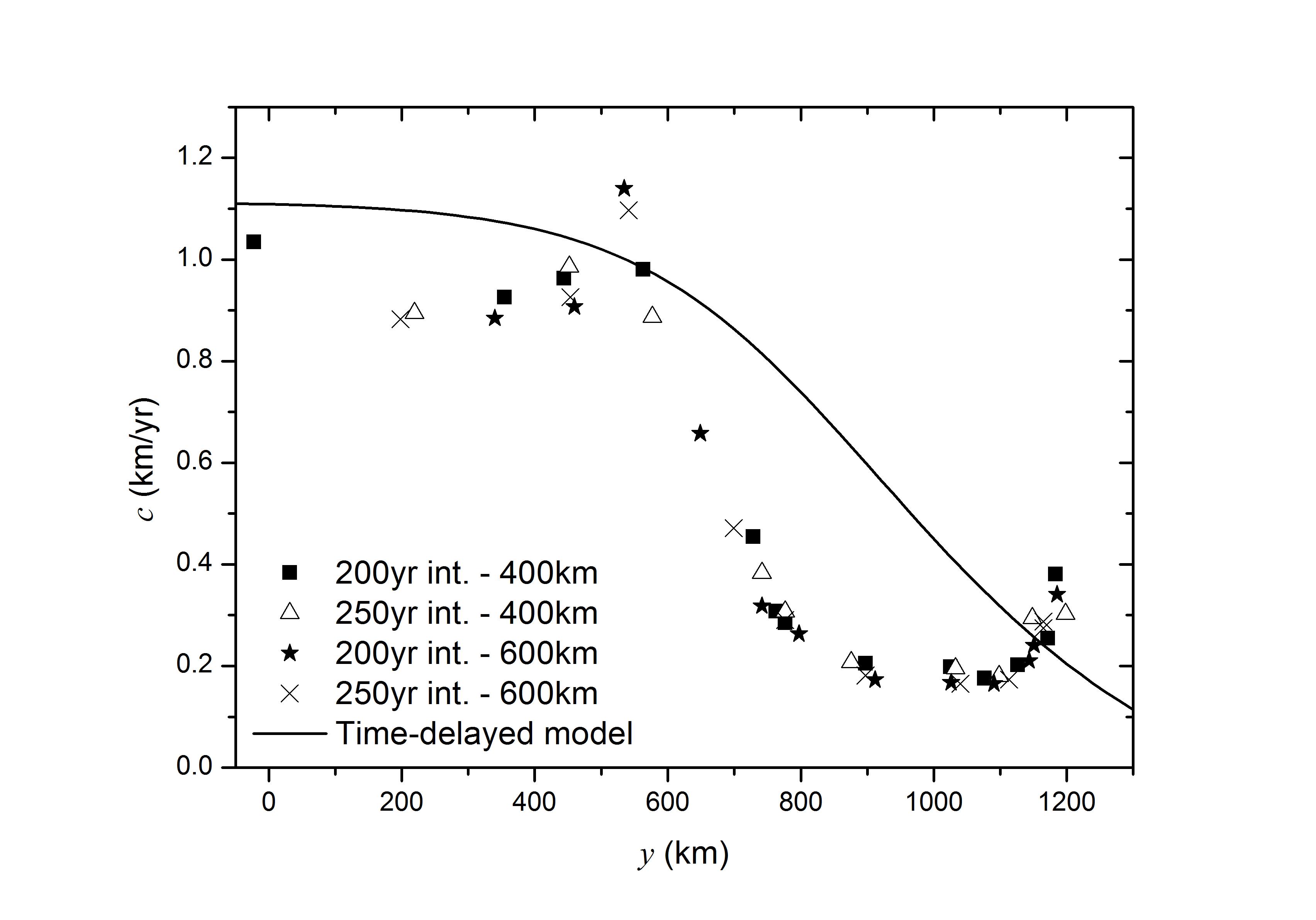

Supplement: Figure S1 — Front speeds estimated from the archaeological data interpolated with a natural neighbor method and using (squares, as in Figs. 2b, 3a–b) and (triangles) intervals for a corridor (see Fig. 1) wide, and (stars) and (crosses) intervals for a corridor wide. The solid line corresponds to the delayed model with space competition (Eq. (1)), i.e., it is the same as the solid line in Figs. 2b and 3a. (TIF) [file pone.0051106.s002.tif]

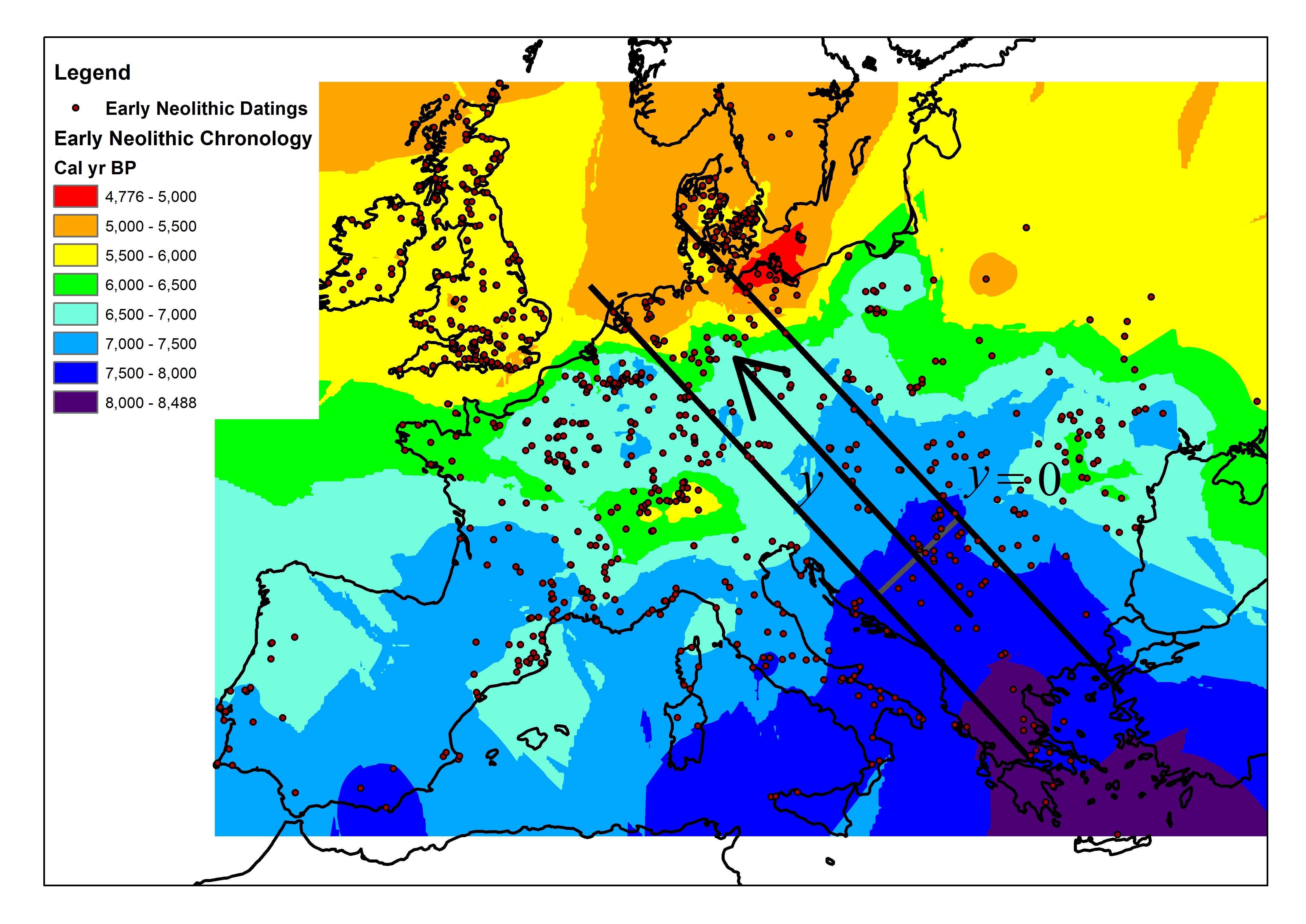

Supplement: Figure S2 — Chronology of the Neolithic expansion calculated with a kringing interpolation method with a spheric semivariogram. The circles correspond to the 902 datings used for this interpolation. The delimited corridor defines the region studied here, where the Neolithic expansion took place mainly in the direction . The origin of the coordinate is also defined on the map. (TIF) [file pone.0051106.s003.tif]

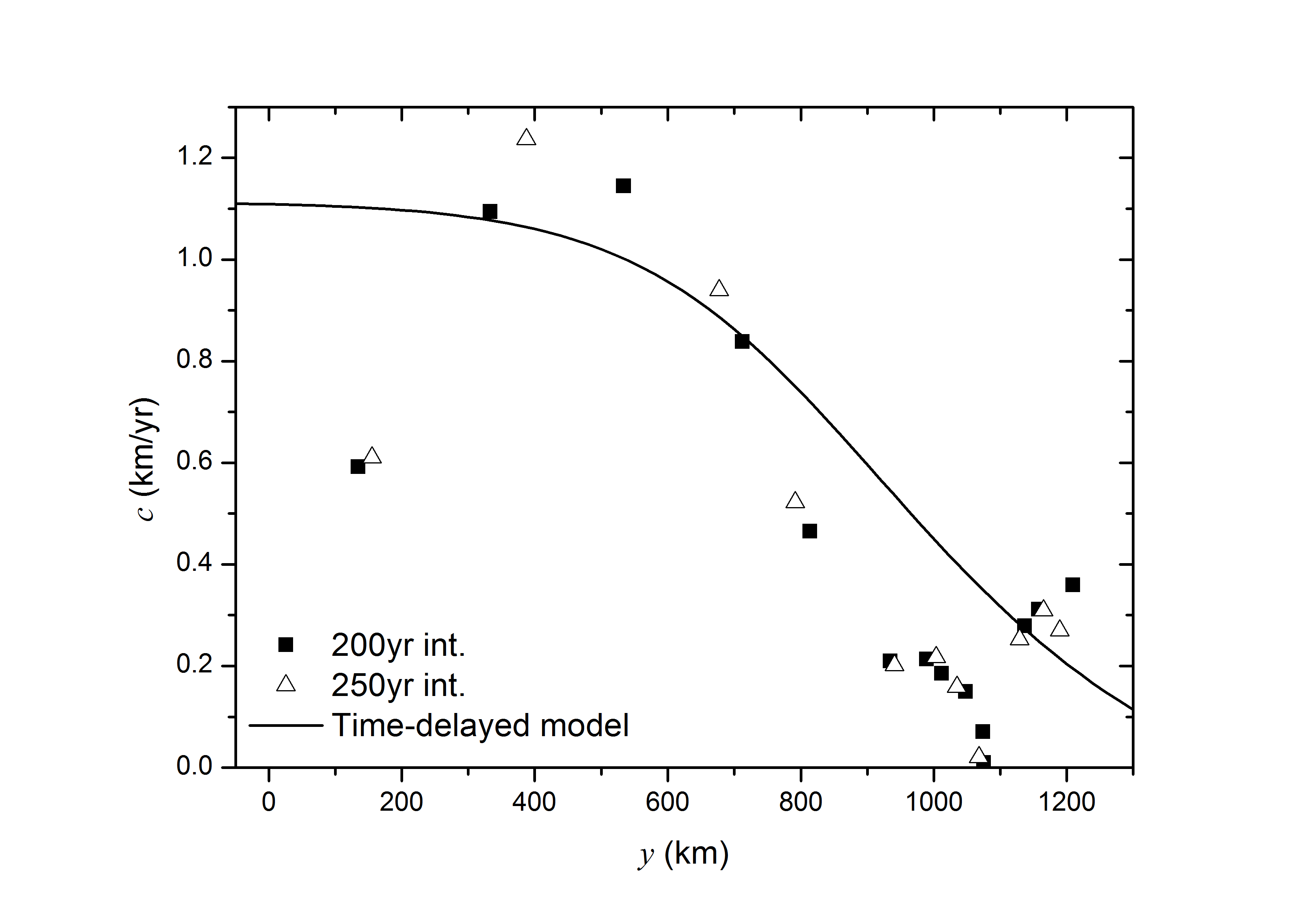

Supplement: Figure S3 — Front speeds estimated from the archaeological data interpolated with a kringing method and using (squares) and (triangles) intervals for a corridor wide (see Fig. 1). The solid line corresponds to the delayed model with space competition (Eq. (1)), i.e., it is the same as the solid line in Figs. 2b and 3a. (TIF) [file pone.0051106.s004.tif]
